# Homelessness and Severe Mental Illness: Challenges and Solutions

**DOI:** 10.7759/cureus.99570

**Published:** 2025-12-18

**Authors:** Saleha Qasim, Luba Leontieva

**Affiliations:** 1 Psychiatry, State University of New York Upstate Medical University, Syracuse, USA

**Keywords:** asylums, deinstitutionalization, homelessness, public health policy, revolving door phenomenon, severe mental illness, state psychiatric hospitals, supportive housing, treatment resistant schizophrenia

## Abstract

Optimal care of people with severe mental illness has always been a challenge. Deinstitutionalization in the second half of the twentieth century paved the way for community programs to help improve the lives of people with mental illness. However, a significant minority of people with severe mental illness continue to fail community housing and rehabilitative services and are falling through the cracks. They often end up in jails, prisons, streets, long-term inpatient psychiatric units, or are frequently admitted to acute inpatient units, leading to the revolving door phenomenon. This paper discusses the current challenges and shortcomings of the available programs for people with severe mental illness and proposes alternative care options for those who fall through the cracks. We propose to establish supportive housing attached to the long-term inpatient units for people with severe mental illness who fail other community programs.

## Editorial

Introduction

According to the National Institute of Mental Health, "Serious mental illness (SMI) is defined as a mental, behavioral, or emotional disorder resulting in serious functional impairment, which substantially interferes with or limits one or more major life activities" [1,2].

Oftentimes in psychiatric practice, professionals encounter a challenging problem of severe mental illness and homelessness. Throughout the history of psychiatry, different solutions have been proposed to optimize the lives of people with severe mental illness, including psychosis. From the building of Kirkbride Asylums to deinstitutionalization and investment in community programs, including Assertive Community Treatment (ACT) [3], Single Room Occupancies (SRO) [4], Supportive apartment programs [5], and Housing First [6], we have come a long way. However, despite all the success that we have had so far, a proportion of the target population continues to suffer, as none of the existing programs suit their needs [1]. This article discusses the shortcomings of the current system and proposes possible alternative solutions and areas to study.

Historical background

Society’s attitude toward people with severe mental illness has evolved over the past few centuries [7]. From leaving them in the community unless they were deemed to be dangerous, followed by confining them in workhouses, madhouses, and state asylums, to shifting the care back to community living as part of “deinstitutionalization”, we have come a long way [7,8].

In early history, people with severe mental illness were left at liberty unless they were deemed dangerous. Later, confinement began, initially with criminals, vagrants, and the idle, in workhouses and houses of correction. It was followed by confinement of the mentally ill to private madhouses and then the establishment of public hospitals and wards for lunatics [7]. One of the rationales for the later confinements was to provide them “asylum” from the dangers of the outside world for their own care and treatment. The conceptual shift to the idea that severe mental illness is treatable by compassionate care and the introduction of the term “moral treatment” led to another wave of institutional establishment. Different types of institutions in different cultures were meant to provide the said compassionate asylum, or in some cases, medical or spiritual treatment for mental illness in different cultures, namely, healing sanctuaries [9,10] and *Bimaristans* (sophisticated, multi-functional institutions that included specialized wards for various diseases, as well as dedicated areas for education, administration, and patient support) in Islamic cultures [11-14].

In the United States, Kirkbride asylums served the purpose and later set the stage for deinstitutionalization. To set the people with mental illness free from the confinements of basements and to treat them, Kirkbride developed the plan for building 'hospitals for the insane': the asylums [15,16]. The Kirkbride asylums were V-shaped buildings with a series of wards or 'pavilions' surrounded by vast gardens. The buildings were designed so that one could view the outside gardens from the tall windows in all the pavilions. These 'asylums' offered a structured living and a healthy lifestyle that was hypothesized to treat people with mental illness. It was supposed to be a 'Victorian Home' for the inhabitants. They had ballrooms, bowling alleys, and other recreational spaces, and the inhabitants worked to aid the functioning of the asylums. Irrespective of the treatment rate, these places were a heaven for the mentally ill and were indeed places for asylum [15,16]. One such example is The Willard Asylum for the chronically insane in Upstate New York [17,18].

The following series of events changed the situation. Though the Kirkbride Asylums were meant to treat the patients, many of the inhabitants never left the asylums once admitted, and others were re-admitted secondary to relapse of mental illness or inability to fit in the community. With a growing population, new patients required institutionalization, and it was hypothesized that industrialization and increased stress increased the prevalence of mental illness. Hence, more patients were being admitted to the asylums than could be cured, and to accommodate them, the initial structure was altered [16]. While Kirkbride asylums were meant for only 250 people, by the mid-twentieth century, one of the hospitals had 14000 patients at a point. The introduction of somatic treatments, overcrowding, burnout of the staff members, and the inability to sustain the system turned them into the 'asylums' represented in the mainstream media [15,16].

The horror stories of abuse and overcrowding were one of the several factors motivating deinstitutionalization. It aimed at improving the lives of people with mental illness, but at the same time, it meant discontinuation of total care for people with severe mental illness who were not able to advocate for their basic needs and required help for arranging the necessities of life [19].

Community programs 

After deinstitutionalization, a variety of community programs have been developed to ensure housing along with good psychiatric care for patients with severe mental illness. Today, after being discharged from an inpatient unit, they can be referred to different community treatment and housing programs based on the patient’s characteristics and available programs in the area. In the state of New York, Single Point of Access (SPOA) connects people with serious mental illness to appropriate services. The main services include individual apartments, SROs, group homes, and supportive housing programs providing varying levels of care and supervision (Table 1) [20]. All of these facilities have specific eligibility requirements, which include (but are not limited to) treatment adherence, level of functionality depending on the facilities available, and the ability to function in the community with available support.

**Table 1:** Types of OMH Licensed Supportive Housing for people with SMI in the State of New York OMH: Office of Mental Health; SMI: serious mental illness

Type of Supportive OMH Licensed Housing	Brief Description
Apartment, Support Community Residence	Apartments in the community with scheduled staff visits for supportive services
Apartment, Treatment Community Residence	Apartments in the community with scheduled staff visits for rehabilitation services
Congregate, Support Community Residence	Group living with 24-hour supervision to maintain and improve functioning
Congregate, Treatment Community Residence	Group living with 24-hour supervision and rehabilitative services
Crisis Residence	A single-site residence with 24-hour supervision for up to 30 days for individuals in acute crisis to prevent re-hospitalization
Service Enriched Single Room Occupancy	Adult private living units (single rooms) at a single site with on-site staff

Additionally, for individuals who are not suitable for housing programs integrated with mental health treatment, Housing First (HF) was developed, as it is argued that homelessness leads to poor mental health outcomes [1]. It is a program that separates the housing and the treatment programs for patients with severe mental illness by moving chronically homeless people with severe psychiatric illness and substance use disorders from the streets into independent apartments rented from community landlords and providing intensive off-site supports [21]. At the same time, the individuals are enrolled in programs like ACT and Intensive Care Management (ICM), but the enrollment is independent of the housing [6,21]. However, there are a minority of patients with severe mental illness and low levels of independent functionality who are not fit for these programs [21]. A study showed that around 13% of people enrolled in HF continue to face housing instability. Among the characteristics found to be more prevalent in these people were the presence of psychotic disorders and chronic homelessness [22].

Although there is not enough evidence to establish the fact, it is observed during clinical practice that patients with severe mental illness and treatment-resistant psychosis are more likely to be the ones who fail these placements. Though attempts at community integration have been successful for most deinstitutionalized patients living in the community who have had better outcomes [23], a minority of the patients have been negatively affected by the movement. The patients with severe mental illness are deemed 'negatively different'. According to a review of multiple articles talking about the effects of deinstitutionalization on the community by Bredewold et al. (2020), deinstitutionalization had both positive and negative effects on the community. The positive effects were seen in the majority in the form of improved quality of life, development and improvement of skills, and better quality of care. The negative effects were seen in several individuals in the form of criminal behavior, victimization, and social isolation. Therefore, the individuals suffering from these “negative effects” are unable to adhere to the community's customs or contribute to it, making them vulnerable to hierarchical and paternalistic dynamics [23]. Even in the community treatment programs, they are at the disposal of healthcare workers and other ‘normals’ of society that control their time, space, and information and resources shared with them. It creates an interpersonal environment dominated by paternalistic and asymmetric relations, minus the symmetrical relations they once had with their fellow inhabitants at the psychiatric hospital [24].

A similar challenge was faced by one of the authors’ patients on a psychiatric unit of an academic hospital in NYS. She was a 59-year-old female with a long history of schizophrenia who presented to the Emergency Department of an academic hospital, disheveled, malodorous, and covered with filth, two days after getting discharged from a long-term inpatient state hospital admission. A detailed review of her core history revealed that she had been hospitalized over 18 times before the reported admission with symptoms of overt psychosis. At the end of every discharge from an inpatient stay, outpatient and community support services were evaluated, and housing support was provided to this patient. She failed multiple group homes, single-room occupancies units, and supportive and independent apartments due to her inability to sustain congregate living (racial slurs, antagonizing others) and unsanitary conditions (she would urinate on the floor, into her clothes, and not take showers or clean after herself). She followed poor hygiene habits during her admission to the acute inpatient psychiatry unit, urinated in bed, and passed racist comments, and continued to disrupt the psychotherapy groups. Given her condition and inability to sustain housing in the past, it was decided that she be transferred back to a long-term inpatient state hospital, as she could not maintain activities of daily living or stay in the supportive or independent housing provided in the past.

Many patients like her continue to go through cycles of inpatient admission, housing placements, treatment failure, relapse, and inpatient hospitalization, leading to a well-known phenomenon named “revolving door”. They are forced to spend their lives on the streets, in homeless shelters, in supervised housing services with limited facilities, in inpatient units far more restricted than asylums, or in jails. The institutions that once provided shelter, food, and a sense of safety are being replaced by subpar alternatives. Unsurprisingly, the number of mentally ill patients in jails and prisons has substantially increased after deinstitutionalization [25]. Multiple studies have discussed a phenomenon called “transinstitutionalization,” characterized by moving people with mental illness from long-term mental health asylums to jails and prisons [26,27]. The Penrose hypothesis, suggested in 1939, that the number of psychiatric beds was inversely related to the size of the prison population [27]. Ashley et al. (2013) estimated the cost of transinstitutionalization in the state of Pennsylvania to be 82.3 million dollars annually, as 6.8% of the year-to-year variation in incarceration rate could be attributed to the decrease in statewide psychiatric beds after controlling for unemployment rate and population growth [26]. It can be argued that residential instability resulting from deinstitutionalization is related to increased arrests of people with mental illness [28]. A randomized trial (N=197) in Canada showed a greater reduction in the rate of arrests (0.36, 95% CI 0.14 to 0.97, p=0.0426) among people with high levels of needs receiving supportive housing (HF and ACT) as compared to the control group who received treatment as usual [29]. An extensive analysis of the data on mental hospitals, jails, and prisons has supported the 'balloon theory', which states that prison and psychiatric hospital populations are inversely related; when one decreases, the other rises [25]. This raises the question of whether we are back where we started in the late eighteenth and early nineteenth centuries, when patients with mental illness spent their lives on streets, in confinement, or in jails. 

Accepting reality

Although efforts related to deinstitutionalization, the development of community programs, and fighting the stigma have alleviated the lives of many people with debilitating psychiatric conditions, there remains a minority of patients who don’t succeed in these programs and continue to suffer even more [23,24]. For them, the goal of community integration proves to be unrealistic because of their severe mental illness affecting their ability to be independent with activities of daily living or to maintain housing [24]. Many community programs provide 24/7 supervision or scheduled visits but are not equipped to assist with activities of daily living [20]. This leads to frequent hospitalizations, higher re-admission rates, increased utilization of emergency rooms and hospital beds. A study describing the typology of hospital trajectories after deinstitutionalization reported that although the severely mentally ill patients or ‘high users’ of service make up only 4.9% of the patients admitted to the psychiatry inpatient unit at a university hospital in Switzerland, they use about a third of hospital beds as calculated by the number of admissions and days of hospital stays over three years period [30].

Many times, they are eventually forced to long-term inpatient units after failing lower levels of care. These units have limited beds and are meant for psychiatric treatment and involve daily rounds by psychiatrists, readily available nursing staff managing the care of patients, therapy groups, and updated treatment plans. All of these services add to the cost of care when the prognosis is not favorable, rendering many of the efforts futile. These resources can be utilized to help those in need who have a more favorable prognosis and can benefit from long-term psychiatric treatment followed by transition to a lower level of care. In addition to the cost of care, these inpatient units are far more restrictive for a person who might be forced to spend the rest of their life there.

Given the above dilemma, we propose that instead of forcing people to either inpatient units or community housing, specialized assisted living facilities associated with psychiatric hospitals be established that should focus on rehabilitation, coping with severe mental illness, and providing support for daily life activities. These facilities should provide a safe space and a home to patients with severe mental illness that is well-suited to their functionality level and allows them to contribute to its working and develop symmetric, healthy relationships with their fellow inhabitants. Such facilities might be close to long-term psychiatric hospitals and operate in conjunction with them. The overall cost will be lower than that of a bed in a psychiatric hospital, as it will not involve daily visits by providers and increased nursing care. The providers can visit periodically, depending on the needs of the residents, and on-site staff with adequate training can manage medications, reducing the number of registered nurses required. It can also be considered as a step-down from the long-term psychiatric hospital (Table 2).

**Table 2:** A comparison of available supportive housing, inpatient services, and the proposed assisted living facilities

Community Housing	Long-Term Inpatient Units	Proposed Assisted Living Facilities
Provide supportive housing in the community for individuals with mental illness and varying levels of functionality.	Inpatient units with defined treatment plans aimed at treating psychiatric conditions and transitioning to a lower level of care, that is, community programs.	Assisted Living Facilities are associated with long-term inpatient units for people with severe mental illness and poor prognosis who fail community housing and require assistance with ADLs.
Lower cost but requires the resident to be able to function in the community to a certain level and be independent with ADLs in most cases.	Much higher cost, as the goal is to treat the mental illness and is meant for people who need a higher level of psychiatric care for a long duration.	Cost will be intermediate as they will require more staffing than the community housing, but will not require daily evaluations by psychiatrists and psychiatric nurses.
Offer more freedom and chances to integrate into the community.	More restricted, limiting the quality of life.	Offers more freedom than inpatient units but is more restricted than community housing.

Conclusion

The above discussion makes it clear that although community treatment and housing programs, as well as long-term inpatient units, successfully provide varying level of psychiatric care and support to people with mental illness, there are a minority of people with severe mental illness and poor prognosis who fail community programs and end up either in prisons, on streets or in the acute or long-term inpatient units. These people need more support and assistance with activities of daily living that community programs cannot provide, and they do not benefit from an inpatient level of psychiatric care, given their prognosis. They are falling through the cracks and need facilities designed to cater to their needs. Alternative approaches that support ADLs and healthcare needs while facilitating patient autonomy and subjective well-being should be explored. This will not only improve the quality of their life and provide the much-needed support but will also be beneficial to the community by reducing the burden on the mental health system, reducing the number of people with SMI in prisons, and preventing the revolving door phenomenon.

## References

[1] Rafla-Yuan E, Handunge VL, White JJ, Castillo EG (2024). Housing, homelessness, and mental health. Psychiatr Ann.

[2] (2025). Mental illness. National Institute of Mental Health (NIMH). https://www.nimh.nih.gov/health/statistics/mental-illness.

[3] Thorning H, Dixon L (2020). Forty-five years later: the challenge of optimizing assertive community treatment. Curr Opin Psychiatry.

[4] Linhorst DM (1991). The use of single room occupancy (SRO) housing as a residential alternative for persons with a chronic mental illness. Community Ment Health J.

[5] Kerman N, Kidd SA, Mutschler C (2023). Managing high-risk behaviours and challenges to prevent housing loss in permanent supportive housing: a rapid review. Harm Reduct J.

[6] Aubry T, Tsemberis S, Adair CE (2015). One-year outcomes of a randomized controlled trial of housing first with ACT in five Canadian cities. Psychiatr Serv.

[7] Parry-Jones WL (2018). Asylum for the mentally ill in historical perspective. BJPsych Bull.

[8] Houston RA (2020). Asylums: the historical perspective before, during, and after. Lancet Psychiatry.

[9] Raguram R, Venkateswaran A, Ramakrishna J, Weiss MG (2002). Traditional community resources for mental health: a report of temple healing from India. BMJ.

[10] Skultans V (1987). The management of mental illness among Maharashtrian families: a case study of a Mahanubhav Healing Temple. Man.

[11] Ahmed JO, Kakamad KK, Najmadden ZB, Saeed SI (2024). Abu Bakr Muhammad ibn Zakariya al-Razi (Rhazes) (865-925): the founder of the first psychiatric ward. Cureus.

[12] Al-Ghazal DSK, El-Gomati M, Abattouy M, Ayduz DS (2025). The origin of bimaristans in Islamic medical history. https://muslimheritage.com/uploads/The_Origin_of_Bimaristans_in_Islamic_Medical_History.pdf.

[13] (2025). Bedlam: the story of Britain's most infamous asylum. History hit. https://www.historyhit.com/bedlam-the-story-of-britains-most-infamous-asylum/.

[14] Daugherty B, Warburton K, Stahl SM (2020). A social history of serious mental illness. CNS Spectr.

[15] Bristow D (2009). Kirkbride's architectural stigma of mental illness. History of Medicine Online.

[16] Allen S, Hall D: The Kirkbride Plan (2019). The Kirkbride plan. https://99percentinvisible.org/episode/the-kirkbride-plan/transcript/.

[17] Doran RE: HISTORY OF WILLARD ASYLUM FOR THE INSANE .AND THE WILLARD STATE HOSPITAL (2025). Willard State Hospital. https://www.asylumprojects.org/index.php?title=Willard_State_Hospital.

[18] Redd W: Inside Willard Asylum (2025). Inside the eerie halls of Willard Asylum, the abandoned New York hospital that's been reclaimed by nature. https://allthatsinteresting.com/willard-asylum.

[19] Bachrach LL (1984). Asylum and chronically ill psychiatric patients. Am J Psychiatry.

[20] (2025). Onondaga County Department of Mental Health. Single point of access (SPOA). https://onondaga.gov/mental-health/spoa/.

[21] Aubry T, Nelson G, Tsemberis S (2015). Housing First for people with severe mental illness who are homeless: a review of the research and findings from the at home--Chez Soi demonstration project. Can J Psychiatry.

[22] Volk JS, Aubry T, Goering P (2016). Tenants with additional needs: when housing first does not solve homelessness. J Ment Health.

[23] Bredewold F, Hermus M, Trappenburg M (2020). ‘Living in the community’ the pros and cons: a systematic literature review of the impact of deinstitutionalisation on people with intellectual and psychiatric disabilities. J Soc Work.

[24] Estroff SE (1981). Psychiatric deinstitutionalization: a sociocultural analysis. J Soc Issues.

[25] (2022). Deinstitutionalization - Special Reports. PBS. https://www.pbs.org/wgbh/pages/frontline/shows/asylums/special/excerpt.html.

[26] Primeau A, Bowers TG, Harrison MA, XuXu XuXu (2013). Deinstitutionalization of the mentally ill: evidence for transinstitutionalization from psychiatric hospitals to penal institutions. Compr Psychol.

[27] Schildbach S, Schildbach C (2018). Criminalization through transinstitutionalization: a critical review of the Penrose hypothesis in the context of compensation imprisonment. Front Psychiatry.

[28] Roy L, Crocker AG, Nicholls TL, Latimer EA, Ayllon AR (2014). Criminal behavior and victimization among homeless individuals with severe mental illness: a systematic review. Psychiatr Serv.

[29] O'Campo P, Stergiopoulos V, Nir P (2016). How did a Housing First intervention improve health and social outcomes among homeless adults with mental illness in Toronto? Two-year outcomes from a randomised trial. BMJ Open.

[30] Golay P, Morandi S, Conus P, Bonsack C (2019). Identifying patterns in psychiatric hospital stays with statistical methods: towards a typology of post-deinstitutionalization hospitalization trajectories. Soc Psychiatry Psychiatr Epidemiol.

